# Encourage the Boys

**Published:** 1875-02

**Authors:** 


ENCOURAGE THE BOYS.—Half-grown boys are too often treated as nuisances, and are thus encouraged to become such. No provision is made for their entertainment; they are not trained to employ their overflowing activity in useful ways; they are treated as if they possessed a peculiar kind of depravity, and as incapable of adding to the happiness of others. It is too much to expect that a boy will prefer reading a dry book to a frolic with his fellows, will enjoy being lectured nightly on his particular sinfulness, while his sister, or some sick, over-studious youth, is held up as a model of perfection. If one half the praise which is bestowed on three and five year olds, and on "young ladies just coming out," were distributed among young men, we should see a marked decline in loaferism and rowdy conduct. To notice a boy's good intention is to make performance easy. To treat him as a social outcast is to make him, sooner or later, a professional disturber of the peace. If home be made pleasant, and pains taken to guide youthful spirits into legitimate channels, there will be less fondness for that independent, roving, selfish existence which marks every boy as an Ishmaelite.

---

HASH is reasonably good food for cats and dogs. It is nearly the worst kind of food for human beings. It is indigestible—not more so, perhaps, than scraps of any other leather, but still, indigestible. For it is leather unquestionably. It is albumen and muscular tissue, originally, as is the somewhat denser sheep-skin or cow-hide, and it is converted into very good leather by the tanning process to which it is subjected. The chopping and stewing hardens the surface of each particle with great skill and certainty, and makes about as abominable a load for the average stomach as can well be devised.

But the worst point about hash is the fact that it is soft; that it is so nearly of the consistency of masticated food, that the tendency is to allow it to slip down the throat unchewed, and without proper salivary mixture. If, after being made with great care, it could be baked hard, dry, and crispy, so that it must be chewed before being swallowed, it would answer quite well as food, though very poorly requiting in actual nutriment, the time and labor demanded in preparing it. But the best use for average hash, is to pass it gracefully over to the cat or dog.

---

A DOMESTIC STORY.—We begin this month the publication of a very entertaining story, entitled "Our Summer at Maplewood," which our lady readers will peruse with interest, and which will prove a very valuable addition to the literature of the household. It will take several months to complete this interesting work, and while each months' installment will possess a value in itself, its attractiveness will be greatly enhanced to those who peruse the chapters consecutively as they appear. The plan of the work, as well as its merit as a literary production, may be fairly determined by the outline given in this number. The writer is a lady of fine literary acquirements, and the methods to be described are such as she has personally tested and found successful.

---

We could pay the national debt in a year if we could annihilate alcohol and tobacco.

